# Esophageal low-grade intraepithelial neoplasia overlying multiple leiomyomas: A case report and review of the literature

**DOI:** 10.3389/fonc.2022.994005

**Published:** 2022-10-31

**Authors:** Wen Pan, Junchao Wu, Chao Liu, Yanjun He, Jinlin Yang

**Affiliations:** ^1^ Department of Gastroenterology and Hepatology, West China Hospital of Sichuan University, Chengdu, China; ^2^ Department of Gastroenterology and Hepatology, The Hospital of Chengdu Office of People’s Government of Tibetan Autonomous Region, Chengdu, China; ^3^ Department of Pathology, The Hospital of Chengdu Office of People’s Government of Tibetan Autonomous Region, Chengdu, China

**Keywords:** esophageal low-grade intraepithelial neoplasia, multiple leiomyomas, overlying, case report, endoscape

## Abstract

**Background:**

Esophageal leiomyoma is the most common benign submucosal mesenchymal tumor. Esophageal intraepithelial neoplasia includes low-grade and high-grade intraepithelial neoplasia. The coexistence of epithelial lesions and the subepithelial lesion is rare. We recorded a case of esophageal low-grade intraepithelial neoplasia (LGIN) overlying multiple esophageal leiomyomas and followed with a review of the literature.

**Case presentation:**

A 49-year-old female patient came for the treatment of esophageal lesions. The submucosal eminences were observed in the right posterior wall and the left anterior wall of the esophagus by Esophagogastroduodenoscopy (EGD). Additionally, we noticed the mucosa of the right wall with brown background color and the dilated, tortuous vessels by narrow-band imaging (NBI). Then we ensured that the submucosal lesions originated from the esophageal mucosal muscle by endoscopic ultrasonography (EUS) and enhanced CT. Subsequently, the submucosal eminence of the right posterior wall and the overlying mucosal lesion were removed together by endoscopic submucosal dissection (ESD). Postoperative pathological diagnosed esophageal submucosal leiomyoma with focal LGIN. Review EGD showed white scars on the right wall of the upper esophagus three months later, while pathological biopsy showed slight squamous epithelial hyperplasia in the left wall. We decided that the left submucosal lesion can be resected at a selective-time operation, and we continue to follow up as planned.

**Conclusions:**

The case of intraepithelial neoplasia overlying the submucosal tumor is rare. Either missed diagnosis or overdiagnosis should be avoided through EGD and pathological biopsy.

## Introduction

The coexistence of epithelial lesion and subepithelial tumor is rare. There were few case reports about the coexistence of esophageal leiomyoma and esophageal severe dysplasia (1–3) and early and advanced (4–7) esophageal squamous cell carcinoma (8–13) since 1987. Some of them were misdiagnosed as advanced esophageal cancer and received surgical operation, while other cases were only diagnosed as esophageal leiomyomas, which were found to be esophageal leiomyomas complicated with early or advanced esophageal cancer by pathological examination after endoscopic or surgical therapy. We recorded a case of esophageal low-grade intraepithelial neoplasia (LGIN) overlying multiple leiomyomas and followed with a review of the literature.

## Case presentation

### Symptoms and personal history

A 49-year-old female patient came to our hospital for the treatment of esophageal high-grade intraepithelial neoplasia overlying subepithelial tumor, which was confirmed at another hospital 4 months ago (**Table 1**). She complained of throat discomfort and mild dysphagia, which appeared after solid food, and without history of family malignancy and cigarette or alcohol use.

**Table 1:** Histopathological features.

Time of sampling	Method of sampling	Location of sampling	Type of staining	Results of pathology
23/07/2021 (another hospital)	Biopsy	Esophagus (20 cm away from the incisor)	HE	Chronic inflammation with focal squamous epithelium HGIN
16/08/2021	Specimen of ESD	Esophagus (the right posterior wall, 18–25c m away from the incisor)	HE	A submucosal spindle cell tumor of focal squamous epithelium with LGIN
		Submucosal tumor (SMT)	SMA	Positive
			Desmin	Positive
		Epithelial layer of mucous membrane	Ki-67	Positive (1%)
22/11/2021	Biopsy	Esophagus (the left wall, 18–21 cm away from the incisor)	HE	The squamous epithelium is mildly hyperplasia

HE, hematoxylin–eosin staining; HGIN, high-grade intraepithelial neoplasia; LGIN, low-grade intraepithelial neoplasia; ESD, endoscopic submucosal dissection; SMA, smooth muscles actin.

### Physical and serological examination

Physical examination findings were typically normal. Serological examination showed no obvious abnormality.

### Esophagogastroduodenoscopy

**Figure 1:**
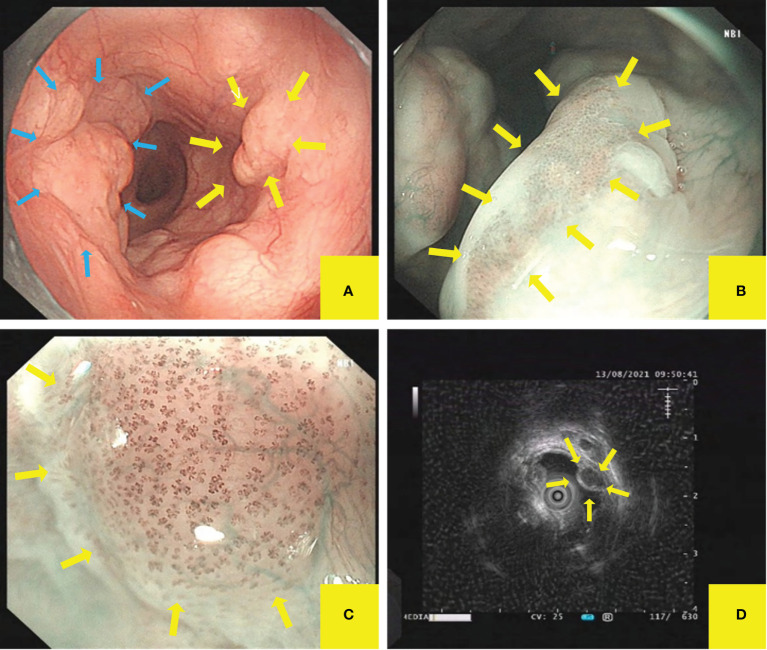
Esophagogastroduodenoscopy (EGD) and endoscopic ultrasonography (EUS) demonstrated esophageal mucosal and submucosal lesions. **(A)** The submucosal eminences were observed in the right posterior wall and the left anterior wall of esophagus. **(B, C)** Multiple shallow red depressions were observed on the right posterior wall surface. The mucosa background color was noticed by NBI, while the dilated and tortuous vessels on the top of the lesion were observed by NBI near focus. **(D)** EUS showed uniform hypoechoic inside, irregularly shaped, clear-boundary esophageal multiple hypoechoic mass, which originated from the mucosal muscle layer.

Esophagogastroduodenoscopy (EGD) demonstrated submucosal eminences in the right posterior wall and the left anterior wall of the esophagus, 18–25 cm away from the incisor teeth, with the widest diameter of about 1.5 cm. Multiple redness and shallow depressions were discovered on the right posterior wall surface additionally (**Figure 1A**). We noticed the mucosa of the right wall with brown background color by using narrow-band imaging (NBI) (**Figure 1B**). Meanwhile, the dilated and tortuous intrapapillary capillary loops with homogeneous distribution and increased density were displayed in the mucosa of the right wall under NBI-near focus (**Figure 1C**). There was no other special finding, such as hiatus hernia, reflux esophagitis, and gastric submucosal lesion on her esophagogastroscopy.

### Endoscopic ultrasonography

We definite the origin and character of the lesion by endoscopic ultrasonography (EUS). We found that esophageal epithelial lesions are clearly stratified, while subepithelial lesion displayed as multiple uniform hypoechoic, irregularly shaped, and clear-boundary masses, which originated from the esophageal mucosal muscle layer and protruded to the cavity inside and outside. The largest one had an ultrasonic diameter of about 9.9×6.9 mm (**Figure 1D**).

### Enhanced chest computed tomography

**Figure 2:**
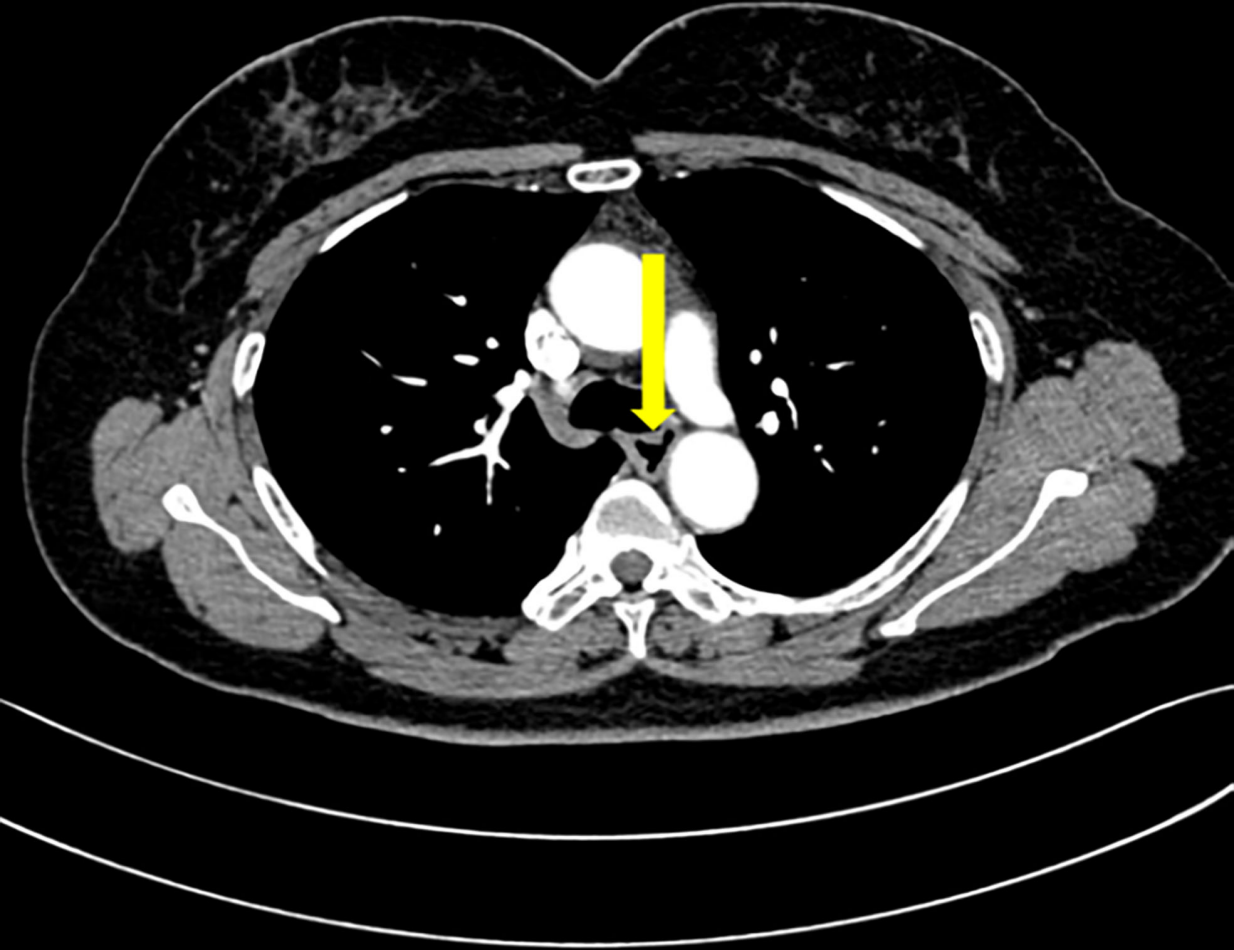
Enhanced chest CT. Enhanced chest CT showed that the wall of the upper and middle segments of the esophagus was slightly thickened and the lumen slightly narrowed.

Enhanced chest CT showed that the wall of the upper and middle segments of the esophagus was slightly thickened and the lumen slightly narrowed (**Figure 2**).

### Treatment and management

#### Endoscopic treatment

**Figure 3:**
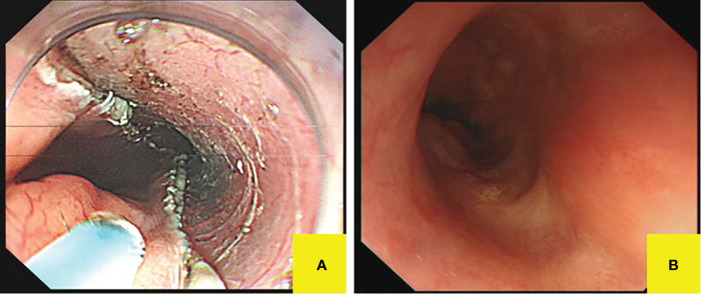
Postoperative endoscopic manifestations. **(A)** The right posterior wall lesion was completely resected without intraoperative complications. **(B)** Follow-up gastroscopy displayed white scars on the right lateral wall of the upper esophagus.

Subsequently, the submucosal eminence of the right wall (18–25 cm away from the incisor) and the overlying mucosal lesion were removed together successfully by ESD without intraoperative complications such as bleeding and perforation, while the submucosal eminence of the left wall was not treated temporarily (**Figure 3A**).

#### Pathohistology

**Figure 4:**
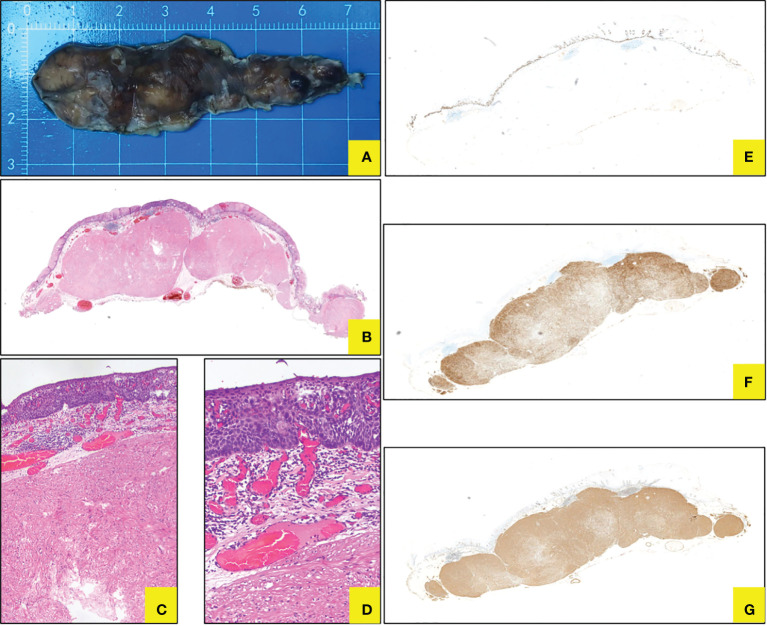
Histopathology for ESD specimen. **(A)** Gross specimen after ESD *in vitro*. **(B–D)** Coexistence of submucosal spindle cell neoplasm and focal squamous LGIN in HE staining (*10, 40, 100). **(E)** Ki76 showed LGIN. **(F)** Desmin staining displayed leiomyoma. **(G)** SMA staining displayed leiomyoma.

We observed the gross specimens after iodine staining and noticed unstained or lightly stained area located mainly at the top of the eminence (**Figure 4A**), which may be related to long-term friction and chronic inflammation. Pathological examination demonstrated submucosal spindle cell tumor with focal squamous epithelium LGIN in the esophagus through hematoxylin–eosin (HE), and finally diagnosed as submucosal leiomyoma with focal LGIN by immunohistochemistry staining (**Table 1** and **Figures 4B–G**).

#### Management of follow-up

The patient was discharged 3 days later and arranged for follow-up 3 months after therapy. When she came back for follow-up, she represented that the symptoms of swallowing obstruction were relieved. Follow-up EGD noticed white scars on the right wall of the upper esophagus (**Figure 3B**), while there was a longitudinal submucosal eminence (18–21 cm away from the incisor) at the left wall, which showed a light brown color change without confirmed demarcation line under NBI. Then, we made the biopsy of the left mucosal lesion, and pathobiology showed slight squamous epithelial hyperplasia in the esophagus (**Table 1**). Then, we made a decision together that the left submucosal lesion can be resected on a selective-time operation, since the esophageal leiomyoma is a benign submucosal tumor and her symptoms have been relieved. We continue to follow up and arranged the next follow-up 1 year later.

## Discussion and conclusions

The coexistence of esophageal leiomyoma and esophageal squamous cell carcinoma can be classified into two types: the overlying type and the separate type (3). The overlying type is rare, let alone multiple leiomyomas covered with esophageal LGIN. To our knowledge, there are no reports about esophageal LGIN overlying multiple leiomyomas before.

Esophageal leiomyoma, which stems from the smooth muscle or muscularis mucosa of the esophagus, is the most common benign submucosal mesenchymal tumor (14, 15). Esophageal leiomyoma can be asymptomatic and found accidentally (7, 9), and the incidence in autopsy series ranges from 0.005% to 5.1% (16, 17). It usually appears as a solitary tumor (97%) (11), and multiple leiomyomas are extremely rare. EGD and EUS can be used to diagnose leiomyoma. EUS can define the layer from which the SMT originates and determine an adequate endoscopic resection strategy (6), which has high diagnostic reliability (18). However, Sheng (19) reported that simple EGD and EUS may miss the diagnosis of leiomyoma combined with esophageal cancer or misdiagnose stromal tumor as leiomyoma. The endoscopic features of esophageal LGIN include mucous membrane turning to red or white, shallow depressions, and obscure or disappearing vessels. The typical pathological manifestations of LGIN are superficially located irregular nuclei, hyperchromatic, mildly enlarged, and nuclei that are oriented perpendicular to the basement membrane without observing the loss of nuclear polarity (20). In our case, the results of EGD, EUS, and biopsy pathological examinations suggested multiple tumors originating from the esophageal mucosal muscle layer and partly covered with LGIN.

Esophageal leiomyoma is considered to be a benign submucosal mesenchymal tumor with asymptomatic complaints unless the tumors are larger than 5 cm in diameter (21, 22). Esophageal leiomyoma has an extremely low possibility of converting into malignancy, and surgical treatment is recommended for tumors that are symptomatic or larger than 5 cm (23, 24). European Society of Gastrointestinal Endoscopy (ESGE) recommends against surveillance of gastrointestinal leiomyomas, provided that these lesions have typical ultrasonographic features (25). However, chronic irritation of the esophageal mucosa caused by intraluminal protrusion of the leiomyoma and esophageal stenosis may also induce or promote malignant transformation in the overlying epithelium (11).

The longest diameter of our patient’s leiomyoma was larger than 5 cm, and the two lesions were located on the contralateral side of the esophagus, resulting in esophageal stenosis, which caused not only the symptoms of swallowing obstruction but also repeatedly friction and inflammatory stimulation of overlying mucosa of leiomyoma, which may be one of the causes of intraepithelial neoplasia.

In 2000, the World Health Organization (WHO) introduced the concept of intraepithelial neoplasia to diagnose precancerous lesions and early cancers of the gastrointestinal tract; LGIN is equivalent to mild and moderate dysplasia, and high-grade intraepithelial neoplasia (HGIN) is equivalent to severe dysplasia and carcinoma *in situ*. As a precancerous disease of esophageal cancer, cigarette and alcohol use are the main risk factors for LGIN (26), and Ki-67 can be used as a marker in histopathology. Our patient did not have the family malignancy history and the habit of cigarette or alcohol use. Her ESD specimen presented the squamous epithelium focal LGIN in HE and immunohistochemistry staining (including Ki-67) (27).

As to management and therapy, small asymptomatic esophageal leiomyomas could be followed up by surveillance (18). When the esophageal leiomyoma is large or symptomatic, and if it originates from the muscularis propria, it is recommended to choose endoscopic resection, which is a safe and effective procedure (6), while esophageal LGIN can also be treated by endoscopic resection or follow-up (28). Compared to surveillance, RFA led the reversion of dysplastic foci to normal epithelium and lower risk of progression to HGIN or carcinoma (29). However, even with careful endoscopic examinations, flat lesions that were considered eligible for RFA might harbor poor histological features, which increase the risk of lymph node metastasis (30). EMR and ESD are safe treatments, whereas ESD is easier than EMR to achieve *en bloc* resection and is appropriate for both epithelial and subepithelial lesions (31).

In our case, RFA is not applicable because of the swelling and uneven surface. Meanwhile, if we resect all of the lesions, there is a high probability of upper esophageal stenosis. Subsequently, we only performed ESD to remove the right lesions to achieve the effect of curing esophageal leiomyoma and LGIN simultaneously. By doing so, we can avoid the deterioration of intraepithelial neoplasia and esophageal stricture after circumferential resection.

In conclusion, the case of mucosal lesion overlying submucosal tumor is rare. Either overdiagnosis or missed diagnosis should be avoided through endoscopic examination and pathological biopsy. Additionally, treatment strategies should be formulated individually to maximize the benefit and minimize the risk for patients.

## Data availability statement

The original contributions presented in the study are included in the article/Supplementary Material. Further inquiries can be directed to the corresponding author.

## Ethics statement

The studies involving human participants were reviewed and approved by the Ethics Committee of The Hospital of Chengdu Office of People’s Government of Tibetan Autonomous Region. The patients/participants provided their written informed consent to participate in this study. Written informed consent was obtained from the individual(s) for the publication of any potentially identifiable images or data included in this article.

## Author contributions

WP, JW, CL, YH, JY conceived and designed the study, and were responsible for the final decision to submit for publication. All authors were involved in the development, review, and approval of the manuscript. All authors contributed to the article and approved the submitted version.

## Funding

This research is funded by Sichuan Medical Association Digestive Endoscopy Special Committee (Jiexiang) Special Research Project (No: 2021XHNJ35)

## Conflict of interest

The authors declare that the research was conducted in the absence of any commercial or financial relationships that could be construed as a potential conflict of interest.

## Publisher’s note

All claims expressed in this article are solely those of the authors and do not necessarily represent those of their affiliated organizations, or those of the publisher, the editors and the reviewers. Any product that may be evaluated in this article, or claim that may be made by its manufacturer, is not guaranteed or endorsed by the publisher.

## Abbreviations

EGD, esophagogastroduodenoscopy; EUS, endoscopic ultrasonography; IEN, Intraepithelial neoplasia; LGIN, low-grade intraepithelial neoplasia; RFA, radiofrequency ablation; EMR, endoscopic mucosal resection; ESD, endoscopic submucosal dissection; NBI, narrow-band imaging.
